# A Comparison of Merkel Cell Carcinoma and Melanoma: Results from the California Cancer Registry

**DOI:** 10.4137/cmo.s423

**Published:** 2008-04-01

**Authors:** Julia Grabowski, Sidney L Saltzstein, Georgia Robins Sadler, Zunera Tahir, Sarah Blair

**Affiliations:** 1 Department of Family and Preventive Medicine, University of California at San Diego School of Medicine, La Jolla, CA; 2 Department of Pathology, University of California at San Diego School of Medicine, La Jolla, CA; 3 Department of Surgery, University of California at San Diego School of Medicine, La Jolla, CA; 4 Rebecca and John Moores UCSD Cancer Center, University of California at San Diego School of Medicine, La Jolla, CA

**Keywords:** merkel cell carcinoma, melanoma, california cancer registry, skin cancer

## Abstract

**Introduction:**

Melanoma and Merkel cell carcinoma (MCC) are both aggressive skin malignancies associated with immunosuppression and possible UV exposure. Both tumors get similar surgical treatment; however, MCC is a relatively rare tumor in which less is known about prognosis and clinical behavior.

**Methods:**

The California Cancer Registry (CCR), a population-based registry, was reviewed from the years 1988–2003. Merkel cell carcinoma and melanoma were compared with relation to gender, age, ethnicity, disease stage, site, and survival.

**Results:**

A total of 113,187 cases of melanoma and 1,878 cases of MCC were identified in the CCR. Though both cancers are more common in men than in women, MCC had a higher incidence in men than melanoma (63% vs 57% p < 0.005). MCC occurs in the more elderly, with 73.6% of cases occurring in people over 70 years. In contrast, 69% of melanoma cases occurred in people younger than 70 years (p < 0.005). MCC shows a predilection for the head and neck compared to melanoma (47% vs 25.8%) Additionally, melanoma occurs more frequently on the trunk than MCC (30% vs 8.7%). Finally, the 10-year cumulative survival is lower for MCC than for melanoma (17.7% vs 61.3%, p < 0.005).

**Conclusion:**

Many clinicians assume MCC and melanoma behave similarly. However, MCC occurs in an older population, more frequently on the head and neck, in a higher percentage of men. Additionally, MCC has a higher rate of regional metastasis and thus may have more of a benefit from regional staging procedures. Overall, MCC has a worse prognosis.

## Introduction

Melanoma and Merkel cell carcinoma (MCC) are both aggressive skin malignancies associated with immunosuppression and UV exposure. Merkel cell carcinoma, unlike melanoma, is exceedingly rare and relatively little is known about its epidemiology and prognosis. Because there are some clear similarities between the diseases and most clinicians have more experience and familiarity with melanoma, MCC has been considered and managed like melanoma.

The Merkel cell was first described in 1875 by Freidrich Merkel. Like the melanocyte, the precursor to melanoma, it is a cell of neuroendocrine origin (Poulson, 2004). It is thought to be derived from the neural crest and migrates to the basal layer of the epidermis where it becomes a slow-acting mechano-receptor. While the first cases of melanoma were described well over 300 years ago, the first cases of MCC were not diagnosed until 1972, when they were called “trabecular carcinoma of the skin.” (Holly et al. 1995; Poulson, 2004). It was later noted, in 1978, that these were tumors of the Merkel cell. Histologically, the tumor is a small-blue cell tumor which can resemble small-cell lung cancer, Ewing’s sarcoma or neuroblastoma (Poulson, 2004). It remains a rare tumor, and based on the most recent epidemiologic data available, the incidence of MCC is only 0.44/100,000 people within the U.S., while the incidence of melanoma is over 20/100,000 (Hodgson, 2005).

The primary treatment for both tumors is surgical excision. At present in the literature, there is level one data on the treatment of melanoma because it is a common tumor and easy to study; however, there is a paucity of data available on even the natural history of MCC. This study compares these two skin based tumors that are treated in a similar surgical fashion but have very a different prognosis. It was designed to examine the behavior of MCC using a population-based cohort in order to highlight some of its distinct characteristics as compared to melanoma.

## Methods

Material from the California Cancer Registry (CCR), a total population-based database, was reviewed from the years 1988 to 2003. The CCR (originally the California Tumor Registry) was established in the late 1940’s as a 10% sample of the incident cancer cases in the state. In 1988, cancer became mandatory reportable disease within California, and continues to be to this the present. All cancers are reported except basal and squamous cell carcinoma of the skin and, since the mid-1990’s, cervical carcinoma in-situ and “borderline” tumors of the ovary. The database includes information about cancer type (histology), patient demographics, disease stage at diagnosis and survival.

All Merkel cell carcinoma and melanoma cases were analyzed with relation to patient gender, ethnicity and age at presentation. Additionally, tumor location and disease stage were examined. For both MCC and melanoma, tumor stage was reported in the CCR as localized, regional, or metastatic. A Berkson-Gage life table was used to calculate cumulative survival and relative cumulative survival. Relative survival rates compare the mortality of a group of patients to a group of people from the general population unaffected by the disease process in-question (Arias, 2002; Cronin and Feuer, 2000). Nominal data were compared using a chi-square.

The CCR does not contain complete information regarding treatment modality for all years so treatment data was not included in our study. Median follow-up was 5 years.

## Results

### Patient and tumor characteristics

A total of 1,878 cases of MCC and 113,187 cases of melanoma were identified. The median age of the MCC patients was 75.5 years (range 24–105 years), with 73.6% of cases occurring in patients over 70 years old. The median age for the melanoma patients was 57 years with a range of 0–107 years (p < 0.05); 69.0% of cases occurred in patients younger than 70 years old. There was a significantly higher percentage of men with MCC than with melanoma (63% vs 57%, p < 0.005). (See Table 1)

MCC and melanoma occur in different locations. MCC and melanoma present with an equal frequency on the extremities (34.7% vs 39.7%, NS). MCC shows a predilection for the head and neck (47.1%), with only 25.8% of melanoma cases presenting in these areas (p < 0.005). Melanoma occurs more frequently on the trunk (30.2%) than does MCC (8.7%, p < 0.005).

For both MCC and melanoma, tumor stage was “unreported” in some cases (26.0% and 19.2%, respectively). The majority of cases of both MCC and melanoma presented with a localized lesion. Melanoma, however, had a higher percentage of patients with localized disease than MCC (75.5% vs 55.8%, p < 0.05). MCC presented at an advanced stage at a higher rate than melanoma. Regional disease, with lymph node involvement, was present in 11.0% of MCC cases and in only 3.1% of melanoma cases (p < 0.05). Metastases were noted in 7.2% of MCC cases, whereas only 2.1% of melanoma cases were metastatic at the time of presentation (p < 0.05).

Both MCC and melanoma were most common in Caucasians (89.1% and 86.7%, respectively), and occurred very rarely in African-Americans (0.96% and 0.35%, respectively) or Hispanics (4.8% and 4.7%, respectively). The rate of MCC and melanoma in Caucasians was significantly higher than the percentage of Caucasians in the California population (51%, p < 0.001) and lower than the percentage of African-Americans and Hispanics in the state (6.9% and 29.9%, p < 0.001). (See Table 2)

### Survival

**Figure 1:**
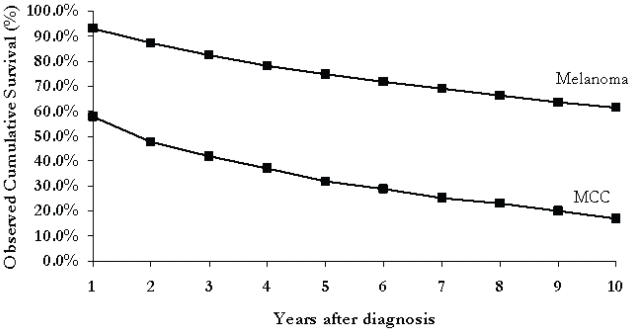


**Figure 2:**
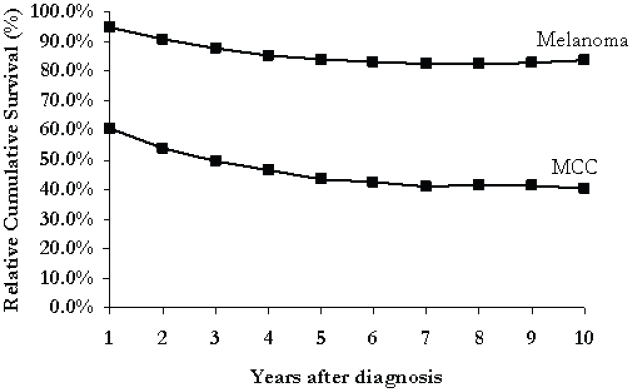


Figure 1 illustrates the cumulative survival rates for MCC and melanoma. After one year, the survival rate is 92.9% for patients with melanoma and only 57.7% for those with MCC (p < 0.05). After 10 years, the cumulative survival from melanoma is 61.3% compared to only 17.7% from MCC (p < 0.05). The relative cumulative survival from melanoma was 94.7% at one year and 83.7% after 10 years. MCC patients had a relative cumulative survival of 60.6% after 1 year and 40.2% after 10 years. (See Fig. 2)

## Discussion

Our review of the literature review suggests that this is the largest series of Merkel cell carcinoma to date with an analysis of over 1,800 cases. Because of its rarity, most of the previously published reports are in the form of small series and case reports with limited conclusions about the natural history and long-term outcomes from MCC.

Merkel cell carcinoma has been perceived as a disease of older men (Brown et al. 1999; Brissett et al. 2002; Koljonen, 2006; Manor, Bellaiche and Bodner, 2006). Indeed, in this study, the majority of patients with both MCC and melanoma were male, however there was a significantly higher percentage of men with MCC. Both melanoma and MCC patients presented in a large range of ages, although only melanoma was noted in children and young adults. The majority of melanoma cases occurred in patients younger than 70 years old while most patients with MCC were older than 70.

Melanoma has long been associated with UV exposure and numerous studies have illustrated a link between sun exposure and disease incidence (Marmelzat, 1977; Holman and Armstrong, 1984; Auteier et al. 1994). The cause of MCC has not been elucidated, though UV exposure has been proposed as a causative factor. A study by Miller and Rabkin found a statistical significance between solar UVB index (annual exposure to UVB radiation) and MCC cases (Miller and Rabkin, 1999). As the CCR contains data only from California, where there is a relatively uniform UVB index throughout the state, it was not possible to draw a conclusion regarding solar index and MCC incidence. However, given that MCC has a higher propensity for sun-exposed areas, such as the head and neck region, this data suggest a possible correlation between MCC and UV exposure. Additionally, the ethnic distribution of both melanoma and MCC seen in the CCR supports the association between UV exposure and these malignancies, as they occur less frequently in highly pigmented groups.

MCC tends to present at a more advanced stage than melanoma. This may be explained in several ways. There may be a difference in the rates of early detection. Melanoma usually presents as an abnormal appearing mole or an atypical nevus which is easily recognizable by clinicians. Education programs have been created and tested to provide community-based melanoma education (Robinson, 1990; Kamin, O’Neill, and Ahearn, 1993). Educational programs have been created to ensure that physicians and nurses are optimally trained to identify lesions and to help educate their patients to do so as well (Gerbert et al. 2002; Berwick et al. 2000). Melanoma’s tendency to occur with greater frequencies in families has prompted providers to focus on education the entire family once one member is diagnosed with melanoma (Hay et al. 2005). Because of the rarity of the tumor, such programs have not been established for MCC, and the tumor is not as easily identifiable. The nodule usually presents as a small, red or violet nodule that may resemble other benign cutaneous lesions and can be overlooked until it gets to an advanced stage (Wong et al. 1998; Poulson, 2004).

Many studies point to Merkel cell carcinoma’s aggressive tumor biology and tendency for early metastases, which may also play a part in the higher proportion of advanced staged disease. In a study by Wong et al., 82% of MCC cases developed metastases at a mean of only 13.4 months after diagnosis of the primary lesion (Wong, 1998). Our study shows an 11.0% rate of regional disease at the time of diagnosis. Though this is significantly higher than the rate seen in melanoma, it is lower than the rates reported in other studies. Several series about MCC showed lymph node involvement in over 20% of patients (Akhtar, Oza, and Wright, 2000; Eng et al. 2004). Disease stage was not reported in 26% of MCC cases within the CCR which may explain the lower rate of regional and metastatic disease in the CCR. Additionally, some cases that had no clinical evidence of metastasis may not have had full work-up for the presence of regional or distant disease involvement and may have been reported as localized without full investigation of tumor stage. The largest single institutional experience in treating MCC is from Memorial Sloan Kettering in which they treated 250 patients over 30 years. In their series, 70 patients with clinically negative regional beds underwent regional staging with either an elective lymph node dissection or sentinel lymph node biopsy. They found that 16 patients (23%) had positive nodes. Therefore, it is likely that many patients in the CCR may be understaged (Allen et al. 2005).

Because of the rarity of the tumor and the overall poor prognosis of MCC, there is little survival data beyond 3 years reported in the literature. In small, retrospective studies, the 2-year survival rate was between 30%–50% (Koljonen, 2006). In contrast, the CCR contains long-term survival data, and analysis of this data confirm the low overall survival rate from MCC, with only a 17.7% cumulative survival after 10 years. Most patients with MCC are elderly, and the low survival rate could be thought to be influenced by their increased age and consequent comorbities. The relative cumulative survival rate, however, compares the mortality of these patients to people from the general population with equivalent ages and comorbidities. The decreased relative survival rate, therefore, should only reflect the impact of MCC. At 10 years, the cumulative survival for people with MCC is only 40.2% of that of the general population.

Given the poor outcomes from MCC, there has been an effort to improve treatment options. MCC, like melanoma, is treated surgically with wide local excision. Unlike melanoma, however, there is limited data on whether sentinel lymph node biopsy is beneficial in MCC. Morton et al. recently showed, in a prospective randomized trial, that patients with intermediate thickness melanoma who underwent a lymph node staging and subsequent lymph node dissection had an improved survival compared to patients who were observed (Morton et al. 2006). Because of the rarity of the tumor, such a study has been difficult to complete for MCC, but there is data to suggest that SLN biopsy may prove valuable for Merkel cell carcinoma patients as well. In the Memorial series, pathologic staging of the draining nodal basin was associated with improved stage-specific survival and decreased nodal recurrence (Allen et al. 2005). Similarly, a recent study reviewed a single institution’s experience as well as small reports from the literature and showed that patients with MCC who had a positive sentinel lymph node biopsy and received adjuvant treatment had an improved survival compared to patients who did not receive adjuvant treatment (Morton et al. 2006). Furthermore, a study focused on Merkel cell carcinoma of the head and neck showed that all patients who underwent wide local incision of the primary tumor with dissection of the lymphatic drainage basin were alive at 2 years as opposed to 68% who had wide local excision alone (Brissett et al. 2002). Though the recent Memorial study did not show any benefit to adjuvant treatment for patients with regional or widely metastatic disease, they did demonstrate that pathologic nodal status was the only independent predictor of survival (Allen et al. 2005). The data from the CCR in our study shows a lower rate of advanced tumors compared to most studies, which suggests that lymph node and distant metastases may not be evident to all clinicians, and even if sentinel lymph node biopsy is not essential for therapy, it may be valuable for accurate staging and counseling patients about prognosis.

Because the CCR does not contain inclusive information regarding treatment for all years, we were unable to analyze outcomes as they relate to treatment. Several studies, however, have shown improved outcomes with adjuvant radiation especially in patients with regional metastasis. Additionally, as MCC appears histologically similar to small cell carcinoma of the lung, similar chemotherapy regimens have been used in conjunction with radiation in high risk patients and have shown improved outcomes (Poulson et al. 2003). Subsequent studies, however, have failed to show any survival benefit with the use of adjuvant chemotherapy and have not advocated its use. Given the overall low survival seen in our study, further studies developing optimal adjuvant treatment protocols are warranted.

## Conclusion

MCC is a very rare tumor and, with such a low incidence, most clinicians may see only a few cases in their career. Given the aggressive nature of the disease, however, knowledge of its unique characteristics are valuable for patient care. Overall, MCC has fundamental differences from melanoma. It affects an older patient population, in a higher percentage of men and occurs more frequently on UV exposed areas, like the head and neck. MCC has a significantly higher rate of regional metastasis than melanoma, and lymph node status has important prognostic implications and may help guide management. Overall, the prognosis is worse for MCC than melanoma and more studies need to be focused on adjuvant treatments for this aggressive disease.

## Figures and Tables

**Table 1:** Demographics of MCC and melanoma patients: gender, age, site of initial lesion, disease stage at presentation.

	MCC (%) n = 1878	Melanoma (%) n = 113,187	p-value
Gender			
Male	63.0	57.0	p < 0.005
Female	37.0	43.0	p < 0.005
Age			
<70 years	26.4	69.0	p < 0.05
≥70 years	73.6	31	p < 0.05
Site			
Extremities	34.7	39.7	NS
Head/Neck Face	47.1	25.8	p < 0.001
Trunk	8.7	30.2	p < 0.005
Other	9.5	4.3	NS
Stage			
Localized	55.8	75.5	p < 0.05
Regional	11.0	3.1	p < 0.05
Metastatic	7.2	2.1	p < 0.05
Unreported	26	19.2	p < 0.05

**Table 2:** Breakdown of ethnicity in entire California population, Merkel cell carcinoma patients and melanoma patients.

	California (%)	MCC (%)	Melanoma (%)
Ethnicity			
Caucasian	51.3	89.1	86.7
African-American	6.9	0.95	0.35
Hispanic	29.9	4.8	4.7
Asian/Pacific Islander	10.6	0.6	0.65
American Indian/Other/Unknown	1.3	7.4	2.6
